# Comparative Analysis of Albumin-Bilirubin (ALBI) Score and Child-Pugh Score for Prognostic Stratification in Hepatocellular Carcinoma Patients: A Single-Center Experience From North India

**DOI:** 10.7759/cureus.112700

**Published:** 2026-07-15

**Authors:** Varun Goel, Arpit Jain, Nivedita Patnaik, Dharmishtha A Basu, Prateek Gupta, Shaifali Goel, Pankaj Goel, Vineet Talwar, Shivendra Singh

**Affiliations:** 1 Department of Medical Oncology, Rajiv Gandhi Cancer Institute and Research Centre, New Delhi, IND; 2 Department of Pathology, BLK-Max Super Speciality Hospital, New Delhi, IND; 3 Department of Surgical Oncology, Rajiv Gandhi Cancer Institute and Research Centre, New Delhi, IND

**Keywords:** albi score, child-pugh score, hepatocellular carcinoma (hcc), liver function, prognosis, survival

## Abstract

Background

Accurate assessment of hepatic reserve is essential in hepatocellular carcinoma (HCC). While the Child-Pugh (CP) score remains widely used, subjectivity in its parameters limits reproducibility. The Albumin-Bilirubin (ALBI) score offers an objective alternative, but contemporary Indian data comparing the two remain limited.

Aim

To compare the prognostic performance of the Child-Pugh (CP) score, the ALBI score, and the modified ALBI (mALBI) grade in predicting survival outcomes among patients with hepatocellular carcinoma treated at a North Indian tertiary cancer center using an extended follow-up dataset.

Methods

This retrospective cohort included consecutive patients diagnosed with HCC between January 2020 and December 2024. Eligibility required radiologic or histologic HCC and baseline CP class A or B. ALBI and CP scores were calculated at diagnosis. Survival analysis was censored on June 30, 2025, providing mature overall survival (OS) and progression-free survival (PFS) data (median follow-up 24.0 months). Kaplan-Meier methods, Cox regression, Harrell’s C-index, and time-dependent receiver operating characteristic (ROC) analyses were performed.

Results

A total of 210 patients were included. ALBI grade distribution was ALBI-1 in 67 patients (31.9%), ALBI-2 in 125 patients (59.5%), and ALBI-3 in 18 patients (8.6%). Child-Pugh classification showed CP-A in 155 patients (74.0%) and CP-B in 55 patients (26.0%). Median OS for the cohort was 14.2 months (95% CI: 11.8-16.9), and median PFS was 9.5 months (95% CI: 7.8-11.4). ALBI demonstrated superior prognostic discrimination compared with Child-Pugh classification (C-index 0.68 vs. 0.61, p=0.024), while modified ALBI showed the highest discriminatory performance (C-index 0.71). ALBI also identified significant prognostic heterogeneity within CP-A patients that was not captured by Child-Pugh classification.

Conclusion

In this retrospective single-center cohort, the ALBI score demonstrated better prognostic discrimination than the Child-Pugh score, particularly among patients with Child-Pugh class A liver function. These findings support consideration of ALBI as an objective tool for risk stratification in HCC; however, prospective multicenter studies are needed to validate its broader clinical application.

## Introduction

Hepatocellular carcinoma (HCC) is the sixth most prevalent cancer and the third leading cause of cancer-related mortality worldwide, with an estimated 905,677 new cases and 830,180 deaths reported in 2020 [1]. The burden is particularly high in Asian countries, including India, where hepatitis B virus (HBV), hepatitis C virus (HCV), and metabolic dysfunction-associated steatotic liver disease (MASLD) constitute the predominant etiologies [2]. The age-standardized incidence rate in India varies from 0.7 to 7.5 per 100,000 individuals, with evidence of a rising trend and marked geographic heterogeneity [3].

A defining challenge in HCC management is its dual pathology: malignancy arising in the setting of underlying chronic liver disease. Approximately 80-90% of patients with HCC have cirrhosis, making hepatic dysfunction both a determinant of treatment eligibility and an independent competing cause of mortality [4,5]. Therefore, an accurate assessment of liver functional reserve is essential for prognostication and appropriate therapeutic decision-making in HCC.

For over five decades, the Child-Pugh (CP) score has been the cornerstone of liver function assessment in HCC and cirrhosis [6]. It incorporates five parameters-bilirubin, albumin, prothrombin time/INR, ascites, and encephalopathy-classifying patients into CP-A, B, or C categories corresponding to increasing hepatic dysfunction [7]. Despite widespread acceptance and integration into frameworks such as the Barcelona Clinic Liver Cancer (BCLC) staging system [8], the CP classification has well-recognized limitations. Two components (ascites and encephalopathy) are subjective and prone to inter-observer variability, reducing reproducibility and discriminative accuracy [9]. Furthermore, its discriminatory capacity is limited even within CP-A, the subgroup comprising most patients evaluated for curative or locoregional therapies [10].

To address these shortcomings, Johnson et al. introduced the Albumin-Bilirubin (ALBI) score in 2015 as a fully objective alternative for evaluating hepatic reserve in HCC [11]. The ALBI score is derived from serum albumin and bilirubin using the formula [11]:

$ALBI = (\log_{10}(\mathrm{bilirubin}[\mu mol/L]) \times 0.66) - (0.085 \times \mathrm{albumin}[g/L])$.

The score stratifies patients into ALBI grades 1-3, with lower scores indicating better hepatic function [11]. Subsequently, the modified ALBI (mALBI) grade subdivided ALBI-2 into 2a and 2b to enhance prognostic discrimination [12].

The ALBI score has gained increasing international acceptance as an objective measure of hepatic functional reserve. Contemporary clinical practice guidelines and expert consensus recognize its value in prognostic stratification and treatment selection, particularly because it avoids the subjectivity associated with the Child-Pugh classification. Nevertheless, its implementation varies across regions, highlighting the need for additional validation using real-world datasets from diverse populations.

Multiple international studies across diverse treatment settings-including hepatic resection, transarterial chemoembolization (TACE), radiofrequency ablation, and systemic therapy-have validated ALBI as at least comparable and often superior to CP in prognostic performance [13-17]. Importantly, ALBI has shown the ability to identify distinct prognostic subgroups within CP-A patients, offering a more nuanced assessment of hepatic reserve [18,19].

Despite robust global evidence, data from the Indian subcontinent remain limited. Variations in etiology, demographic patterns, disease stage at presentation, and therapeutic practices underscore the need for regional validation studies [20]. Moreover, CP scoring continues to dominate clinical decision-making in many Indian centers, with limited real-world comparative evaluations.

Therefore, this study was undertaken to compare the prognostic performance of the Child-Pugh score, the ALBI score, and the modified ALBI (mALBI) grade in predicting survival outcomes among patients with hepatocellular carcinoma treated at a tertiary cancer center in North India. We hypothesized that ALBI and mALBI would demonstrate superior prognostic discrimination compared with the Child-Pugh score, particularly in identifying prognostic heterogeneity within the Child-Pugh class A subgroup, thereby supporting the use of objective liver function assessment in clinical practice.

## Materials and methods

Study design and patient selection

This retrospective, observational, single-center cohort study was conducted at the Department of Medical Oncology, Rajiv Gandhi Cancer Institute and Research Centre (RGCIRC), New Delhi, India. All patient records were deidentified and anonymized prior to data extraction and statistical analysis. No patient identifiers were accessible to investigators during study conduct. Institutional Review Board, Rajiv Gandhi Cancer Institute and Research Centre (RGCIRC), New Delhi issued approval RES/SCM/70/2025/79, dated December 15, 2025, and informed consent was waived because of the retrospective design.

All consecutive adult patients diagnosed with HCC between January 2020 and December 2024 at RGCIRC, New Delhi, were screened for eligibility. Patients were included if they were aged 18 years or older, had either histologically confirmed HCC or radiological evidence of HCC according to accepted American Association for the Study of Liver Diseases (AASLD) [21] and European Association for the Study of the Liver (EASL) [22] diagnostic criteria, possessed baseline serum albumin and bilirubin measurements obtained within two weeks of diagnosis, and had Child-Pugh class A or B liver function at presentation. Additionally, patients were required to have at least three months of follow-up or documented disease progression or death before that interval.

Patients were excluded if they presented with Child-Pugh class C liver disease, had previously received treatment for HCC at another institution, had a concurrent active malignancy, lacked essential baseline laboratory or imaging data, had undergone prior liver transplantation, or were lost to follow-up within three months without documented disease progression or mortality.

Data collection

Demographic, clinical, laboratory, radiological, and treatment information was extracted from electronic medical records. Baseline characteristics included age, sex, Eastern Cooperative Oncology Group (ECOG) performance status [23], BMI, etiology of liver disease (HBV, HCV, alcohol-related, nonalcoholic fatty liver disease {NAFLD}, or cryptogenic), presence of cirrhosis, and key comorbidities such as diabetes and hypertension.

Laboratory parameters collected within two weeks before treatment initiation included complete blood count, serum albumin, total bilirubin, aspartate aminotransferase (AST), alanine aminotransferase (ALT), alkaline phosphatase (ALP), gamma-glutamyl transferase (GGT), serum creatinine, blood urea nitrogen (BUN), international normalized ratio (INR)/prothrombin time, and serum alpha-fetoprotein (AFP). Radiological tumor assessment was performed using contrast-enhanced CT or MRI. Tumor burden variables included number of lesions, maximum tumor diameter, macrovascular invasion (portal vein or hepatic vein thrombosis, inferior vena cava involvement), extrahepatic spread (lymph nodes, lung, bone, or others), and presence of ascites. Disease stage was categorized using the BCLC staging system (0, A, B, C, D) [24].

Liver function assessment

The Child-Pugh score was calculated using five components: serum albumin, total bilirubin, INR/prothrombin time, ascites (absent/mild/moderate-to-severe), and hepatic encephalopathy (absent/mild-to-moderate/severe) [25]. Each parameter was scored 1-3, generating total scores of 5-15, corresponding to CP-A (5-6), CP-B (7-9), and CP-C (10-15). For subgroup analyses, CP-A patients were further classified as CP-A5 or CP-A6, and CP-B patients as CP-B7, CP-B8, or CP-B9.

The ALBI score was calculated using the established formula [11]:

$ALBI = (\log_{10}(\mathrm{bilirubin}[\mu mol/L]) \times 0.66) - (0.085 \times \mathrm{albumin}[g/L])$.

Bilirubin values measured in mg/dL were converted to μmol/L by multiplying by 17.1, while albumin values were expressed in g/L before ALBI calculation.

Patients were categorized into: (i) ALBI grade 1: ≤ -2.60; (ii) ALBI grade 2: > -2.60 to ≤ -1.39; (iii) ALBI grade 3: > -1.39.

To improve prognostic resolution, modified ALBI (mALBI) grading subdivided ALBI grade 2 into: (i) Grade 2a: > -2.60 to ≤ -2.27; (ii) Grade 2b: > -2.27 to ≤ -1.39 [12].

Treatment and follow-up

Treatment decisions were guided by a multidisciplinary tumor board comprising medical oncology, surgical oncology, hepatobiliary surgery, interventional radiology, and radiation oncology specialists. Management strategies included hepatic resection, radiofrequency ablation (RFA), transarterial chemoembolization (TACE), transarterial radioembolization (TARE), stereotactic body radiotherapy (SBRT), systemic therapy (sorafenib, lenvatinib, immunotherapy combinations), or best supportive care.

Patients underwent regular follow-up with clinical assessments and laboratory tests every four to eight weeks. Imaging with CT or MRI was typically performed every 8-12 weeks. Radiological response was assessed using modified Response Evaluation Criteria in Solid Tumors (mRECIST) for HCC [26]. The follow-up cutoff was June 30, 2025, ensuring ≥6 months of follow-up for all patients diagnosed in 2024.

Outcome measures

The primary outcome was overall survival (OS), defined as the interval from diagnosis to death from any cause or last known follow-up.

The secondary outcome was progression-free survival (PFS), defined as time from diagnosis to radiological progression, death, or last follow-up, whichever occurred earlier.

Survival outcomes were obtained from electronic records, hospital mortality registries, and telephonic follow-up. June 30, 2025, was used as the data cutoff date for survival analyses.

Statistical analysis

Continuous variables were expressed as mean ± standard deviation (SD) or median with interquartile range (IQR) depending on distribution (Shapiro-Wilk test). Comparisons were performed using Student’s t-test or Mann-Whitney U test for continuous variables and chi-square or Fisher’s exact tests for categorical variables.

Survival curves were generated using the Kaplan-Meier method and compared using the log-rank test. Univariate Cox regression identified variables associated with OS and PFS. Variables demonstrating an association with survival in univariate analysis (p < 0.10), together with clinically relevant covariates identified from previous literature, were considered for inclusion in the multivariable Cox regression model. Model selection was performed using a forward stepwise approach to derive a parsimonious model while minimizing overfitting. The proportional hazards assumption for the Cox regression models was assessed using Schoenfeld residuals. Both global and covariate-specific tests were performed. No statistically significant violations of the proportional hazards assumption were observed (all p > 0.05), confirming the appropriateness of the Cox proportional hazards model for survival analyses. Missing baseline data were minimal; therefore, a complete-case analysis was performed. Hazard ratios (HR) with 95% confidence intervals (CI) were reported.

Prognostic performance of CP and ALBI scores was assessed using Harrell’s concordance index (C-index). Differences in C-indices were tested using the DeLong method. Time-dependent receiver operating characteristic (ROC) curves and area under the curve (AUC) values at 6, 12, and 24 months were generated to evaluate discriminatory ability.

Subgroup analyses included prognostic stratification of CP-A patients across ALBI grades and evaluation of CP-A versus CP-B patients within ALBI grade 2. These subgroup and pairwise analyses were prespecified as exploratory analyses. Accordingly, p-values from these analyses were interpreted descriptively, and no formal adjustment for multiple comparisons was performed. All tests were two-sided, with p < 0.05 considered statistically significant. Analyses were performed using IBM SPSS Statistics for Windows, version 26 (IBM Corp., Armonk, NY, USA), and R version 4.2.0 (R Foundation for Statistical Computing, Vienna, Austria) with the survival, survminer, Hmisc, and timeROC packages.

## Results

Patient characteristics

**Figure 1:**
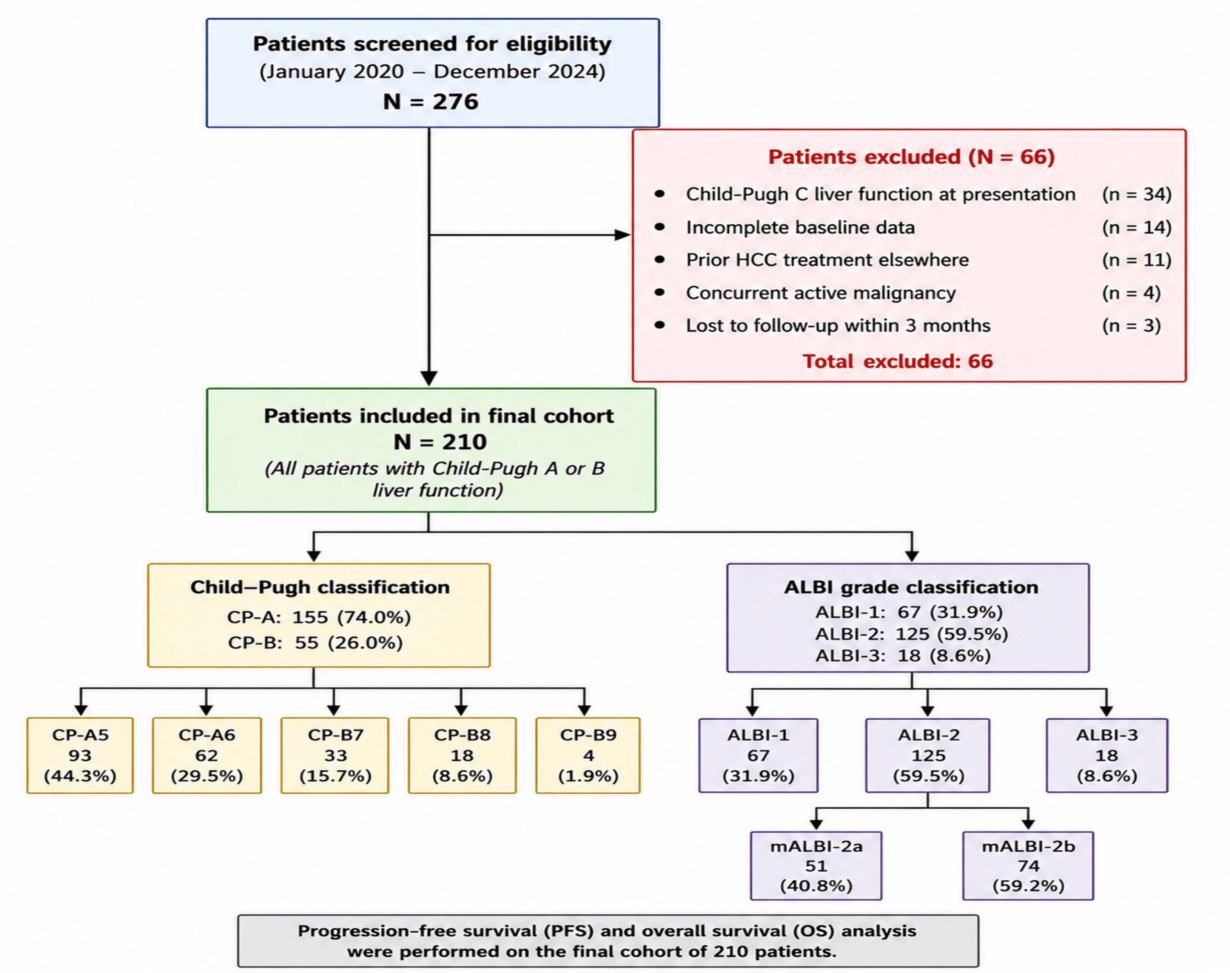
Flow diagram of patient selection and study cohort formation. Flowchart depicting patient screening, exclusions, final cohort inclusion, and stratification according to Child-Pugh (CP) and Albumin-Bilirubin (ALBI) classifications for survival analyses. HCC, hepatocellular carcinoma.

Between January 2020 and December 2024, a total of 276 patients with hepatocellular carcinoma (HCC) were screened. After applying the predefined inclusion and exclusion criteria, 210 patients were included in the final study cohort (Figure 1). A total of 66 patients were excluded because of Child-Pugh class C liver function at presentation (n = 34, 12.3%), incomplete baseline laboratory/imaging data (n = 14, 5.1%), prior HCC treatment at another institution (n = 11, 4.0%), concurrent active malignancy (n = 4, 1.4%), and loss to follow-up within three months (n = 3, 1.1%).

Baseline demographic and clinical characteristics are summarized in Table 1. The cohort had a mean age of 58.7 ± 9.4 years, with 82.4% males (n = 173). An ECOG performance status of 0-1 was observed in 165 patients (78.6%). HBV infection was the predominant etiology (n=91, 43.3%), followed by HCV (n=52, 24.8%), alcohol-related liver disease (n=32, 15.2%), NAFLD (n=19, 9.0%), and cryptogenic cirrhosis (n=16, 7.6%). Underlying cirrhosis was present in 188 patients (89.5%).

**Table 1:** Baseline demographic and clinical characteristics AFP, alpha-fetoprotein; BCLC, Barcelona Clinic Liver Cancer; ECOG PS, Eastern Cooperative Oncology Group performance status; HBV, hepatitis B virus; HCV, hepatitis C virus; IQR, interquartile range; NAFLD, nonalcoholic fatty liver disease.

Characteristic	Value
Age (years), mean ± SD	58.7 ± 9.4
Male gender, n (%)	173 (82.4)
ECOG PS 0–1, n (%)	165 (78.6)
Etiology	
HBV, n (%)	91 (43.3)
HCV, n (%)	52 (24.8)
Alcohol-related liver disease, n (%)	32 (15.2)
NAFLD, n (%)	19 (9.0)
Cryptogenic, n (%)	16 (7.6)
Cirrhosis present, n (%)	188 (89.5)
Laboratory parameters	
Albumin (g/dL), median (IQR)	3.6 (3.2-4.0)
Bilirubin (mg/dL), median (IQR)	1.2 (0.8-1.8)
AFP ≥200 ng/mL, n (%)	122 (58.1)
Tumor characteristics	
Solitary tumor, n (%)	83 (39.5)
Maximum tumor diameter (cm), median (IQR)	6.9 (4.3-9.6)
Macrovascular invasion, n (%)	64 (30.5)
Extrahepatic metastasis, n (%)	39 (18.6)
BCLC stage	
Stage 0-A, n (%)	20 (9.5)
Stage B, n (%)	67 (31.9)
Stage C, n (%)	101 (48.1)
Stage D, n (%)	22 (10.5)

Median albumin and bilirubin levels at diagnosis were 3.6 g/dL (IQR 3.2-4.0) and 1.2 mg/dL (IQR 0.8-1.8), respectively. Elevated AFP ≥200 ng/mL was noted in 122 patients (58.1%). Solitary tumors were observed in 83 patients (39.5%), while 127 (60.5%) had multifocal disease. Median maximum tumor diameter was 6.9 cm (IQR 4.3-9.6). Macrovascular invasion and extrahepatic metastasis were present in 64 (30.5%) and 39 (18.6%) patients, respectively.

According to BCLC staging, 20 (9.5%) patients had stage 0-A disease, 67 (31.9%) had stage B, 101 (48.1%) had stage C, and 22 (10.5%) had stage D. Child-Pugh classification showed that 155 (74.0%) patients were CP-A and 55 (26.0%) were CP-B. Subclassification revealed CP-A5: 93 (44.3%), CP-A6: 62 (29.5%), CP-B7: 33 (15.7%), CP-B8: 18 (8.6%), and CP-B9: 4 (1.9%), reflecting predominantly preserved liver function despite advanced tumor stages.

Distribution of ALBI and Child-Pugh scores

ALBI grading stratified the cohort into ALBI-1: 67 (31.9%), ALBI-2: 125 (59.5%), and ALBI-3: 18 (8.6%). Of the 125 ALBI-2 patients, 51 (40.8%) were classified as mALBI-2a and 74 (59.2%) as mALBI-2b, demonstrating the expected predominance of intermediate ALBI values in routine clinical practice.

Cross-tabulation of ALBI and CP classes demonstrated a statistically significant association (p < 0.001) but also highlighted substantial heterogeneity within CP-A (Table 2). 

**Table 2:** Cross-tabulation of Child-Pugh score and ALBI grade (Pearson χ²=54.8, p<0.001). CP, Child-Pugh; ALBI, Albumin-Bilirubin.

Child-Pugh class	ALBI-1 n (%)	ALBI-2 n (%)	ALBI-3 n (%)	Total n (%)
CP-A5	54 (58.0)	39 (42.0)	0 (0)	93 (44.3)
CP-A6	5 (8.1)	55 (88.7)	2 (3.2)	62 (29.5)
CP-B7	0 (0)	29 (87.9)	4 (12.1)	33 (15.7)
CP-B8	0 (0)	17 (94.4)	1 (5.6)	18 (8.6)
CP-B9	0 (0)	0 (0)	4 (100)	4 (1.9)
Total	67 (31.9)	125 (59.5)	18 (8.6)	210 (100)

**Figure 2:**
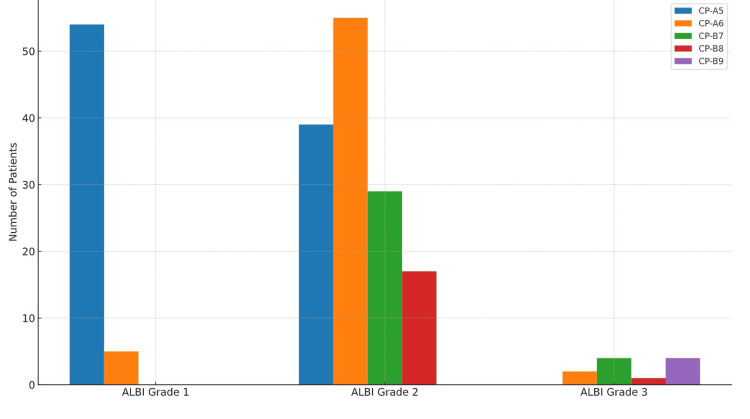
Distribution of ALBI grades across Child-Pugh classes (N = 210). ALBI grading reveals marked heterogeneity within CP-A and CP-B categories, with ALBI providing finer liver function discrimination than Child-Pugh. CP, Child-Pugh; ALBI, Albumin-Bilirubin.

Among CP-A5 patients, 54 of 93 (58.0%) were classified as ALBI-1, compared with 5 of 62 (8.1%) among CP-A6 patients, indicating markedly different functional liver reserve within the CP-A category. All 4 of 4 (100%) CP-B9 patients were classified as ALBI-3, reflecting severely impaired hepatic function. Overall, ALBI provided a more granular separation of liver function than CP (Figure 2).

Treatment distribution

Table 3 displays the treatment modalities delivered. The most frequently used modality was TACE (n=90, 42.8%), followed by systemic therapy (n=68, 32.4%). Sorafenib (n=46, 21.9%) remained the most common systemic agent, followed by lenvatinib (n=16, 7.6%) and atezolizumab-bevacizumab combination therapy (n=11, 5.2%). Curative-intent therapies included hepatic resection (n=23, 11.0%) and RFA (n=13, 6.2%), predominantly administered to ALBI-1 and CP-A5 patients. SBRT was used in 10 patients (4.8%), while best supportive care alone was offered to 3 patients (1.4%).

**Table 3:** Distribution of treatment modalities. TACE, transarterial chemoembolization.

Treatment modality	n (%)
TACE	90 (42.8)
Systemic therapy	68 (32.4)
-Sorafenib	46 (21.9)
-Lenvatinib	16 (7.6)
-Atezolizumab + Bevacizumab	11 (5.2)
Hepatic resection	23 (11.0)
Radiofrequency ablation (RFA)	13 (6.2)
Stereotactic body radiation therapy (SBRT)	10 (4.8)
Best supportive care	3 (1.4)
Total	210 (100)

Treatment allocation differed significantly by ALBI and CP classifications, aligning with their clinical relevance. Patients with poorer ALBI grade or CP class were more frequently managed with non-curative modalities due to diminished hepatic reserve and greater tumor burden.

Survival analysis

**Figure 3:**
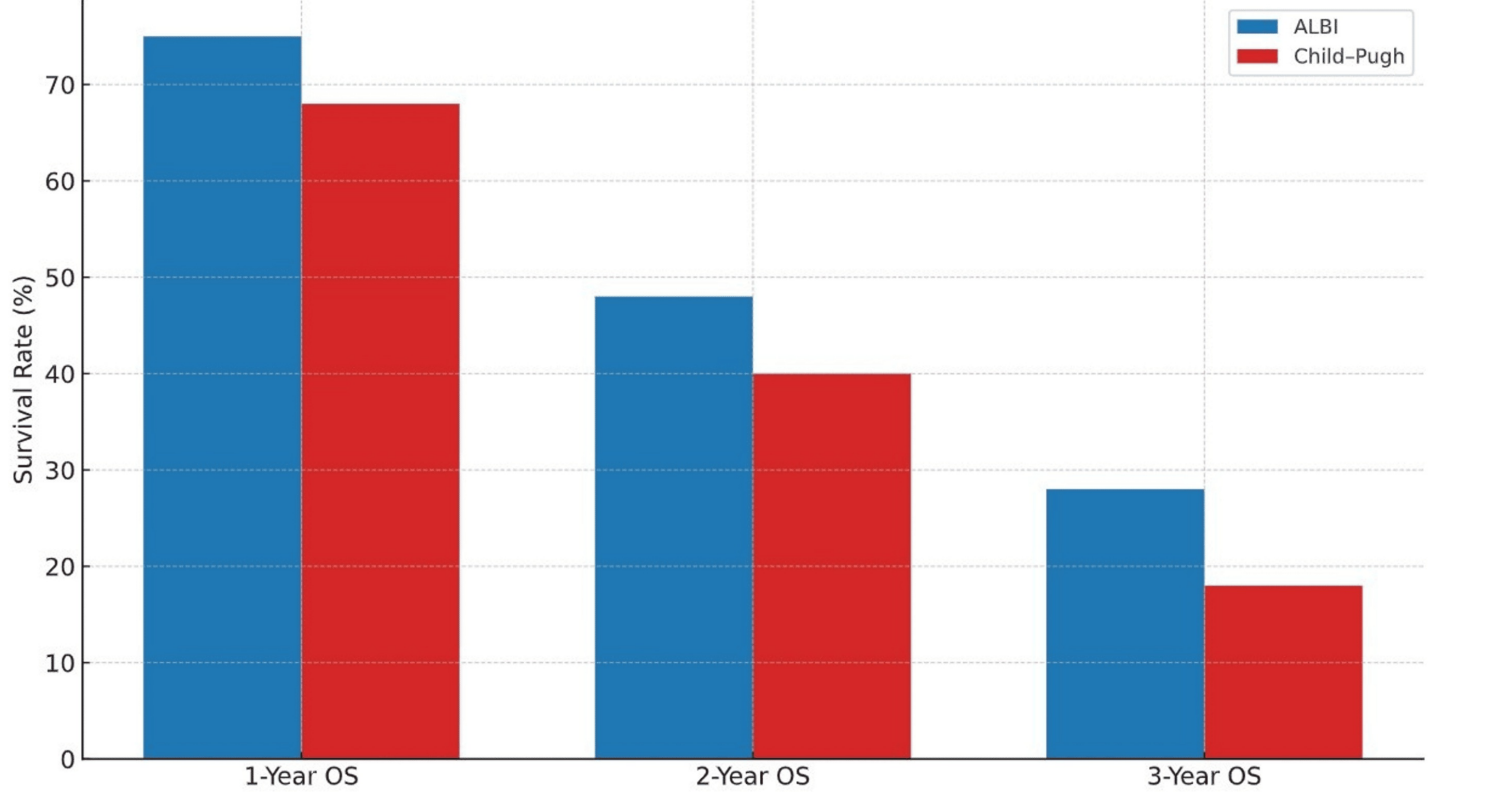
Survival rates by ALBI vs. Child-Pugh. One-, two-, and three-year overall survival rates were consistently higher for ALBI-1 vs. ALBI-2 and ALBI-3, outperforming the Child-Pugh system. ALBI, Albumin-Bilirubin; OS, overall survival.

At the study follow-up cut-off of June 30, 2025, 138 deaths (65.7%) had occurred. The median overall survival (OS) for the entire cohort was 14.2 months (95% CI: 11.8-16.9 months). Patients classified as Child-Pugh class A demonstrated significantly superior survival compared with Child-Pugh class B patients, with median OS values of 16.4 months (95% CI: 13.8-19.2) and 8.7 months (95% CI: 6.2-11.5), respectively (log-rank p<0.001) (Figure 3).

Further subclassification of Child-Pugh class A patients revealed clinically meaningful heterogeneity. Patients with CP-A5 liver function achieved a median OS of 18.9 months, whereas those with CP-A6 liver function had a median OS of 11.8 months (p=0.008). Although survival progressively worsened across CP-B7, CP-B8, and CP-B9 categories, statistical significance was not reached, likely because of limited subgroup sample sizes.

**Figure 4:**
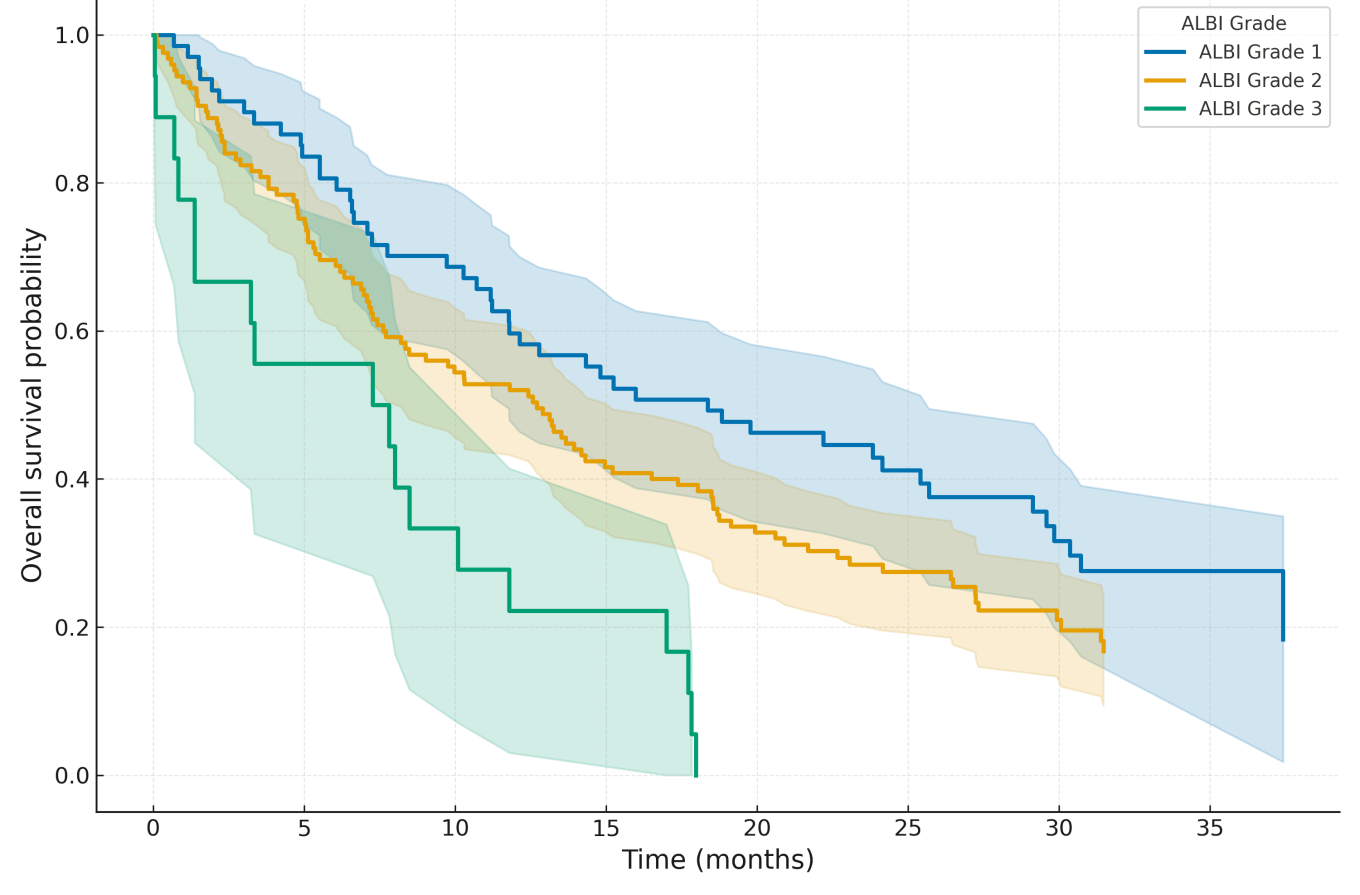
Kaplan-Meier survival by ALBI grade. ALBI grades 1, 2, and 3 show significantly different overall survival curves (log-rank p < 0.001), demonstrating strong prognostic stratification. ALBI, Albumin-Bilirubin.

ALBI grading demonstrated even stronger prognostic discrimination. Median OS was 22.5 months (95% CI: 18.4-27.8) for ALBI grade 1, 12.8 months (95% CI: 10.5-15.3) for ALBI grade 2, and 5.4 months (95% CI: 3.1-8.2) for ALBI grade 3 (log-rank p<0.001). All pairwise comparisons were statistically significant. Furthermore, modified ALBI grading enhanced prognostic resolution, with median OS of 15.6 months for mALBI-2a compared with 10.4 months for mALBI-2b (Figure 4).

Among the 155 patients classified as Child-Pugh class A, ALBI grading identified substantial prognostic heterogeneity. Patients categorized as ALBI-1 within the CP-A subgroup achieved a median OS of 18.6 months (95% CI: 15.2-22.8), whereas those classified as ALBI-2 had a median OS of 12.3 months (95% CI: 9.8-15.1) (p=0.003). The observed 6.3-month difference in median survival highlights the superior discriminatory capability of ALBI in identifying clinically relevant differences in hepatic reserve among patients traditionally considered to have well-compensated liver function.

Comparison of discriminatory performance

The discriminatory performance of both the ALBI score and CP score for predicting overall survival was compared using Harrell's C-index (Table 4).

**Table 4:** Comparison of discriminatory performance (C-index) for overall survival prediction. ALBI, Albumin-Bilirubin.

Scoring system	C-index (95% CI)	p-value
Child-Pugh score	0.61 (0.55-0.67)	Reference
ALBI score	0.68 (0.62-0.74)	0.024
Modified ALBI score	0.71 (0.65-0.77)	0.008

**Figure 5:**
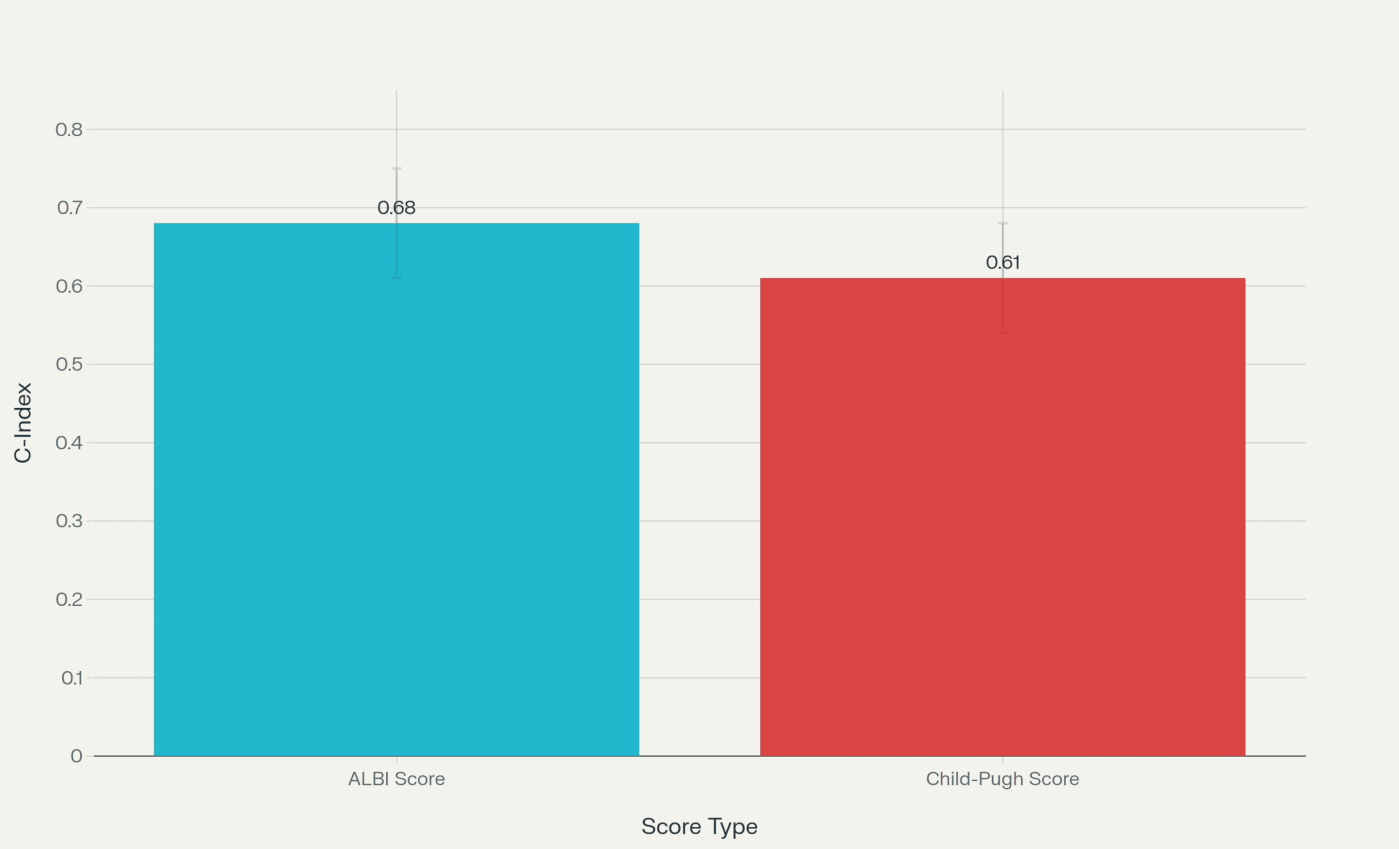
ALBI vs. Child-Pugh discriminatory performance. Comparison of Harrell’s C-index shows ALBI (0.68) outperforming Child-Pugh (0.61) for overall survival prediction (p = 0.024). ALBI, Albumin-Bilirubin.

The ALBI score had a C-index of 0.68 (95% CI: 0.62-0.74), better than the CP score of 0.61 (95% CI: 0.55-0.67); p=0.024. The modified ALBI grading variable had even better discriminatory performance with a C-index of 0.71 (95% CI: 0.65-0.77); p = 0.008 (Figure 5).

The time-dependent ROC analysis at 6 months, 12 months, and 24 months had an area under the curve (AUC) score that was consistently higher for the ALBI score than for the CP score. At 12-month follow-up, the AUC for the ALBI score was 0.72 (95% CI: 0.64-0.80) and for the CP score was 0.65 (95% CI: 0.56-0.74); p=0.031.

Univariate and multivariate analysis

Univariate analysis identified ECOG ≥2, AFP ≥200 ng/mL, multifocal tumors, tumor size >5 cm, macrovascular invasion, extrahepatic metastasis, BCLC stage C-D, CP-B classification, and ALBI ≥2 as significant predictors of survival.

**Figure 6:**
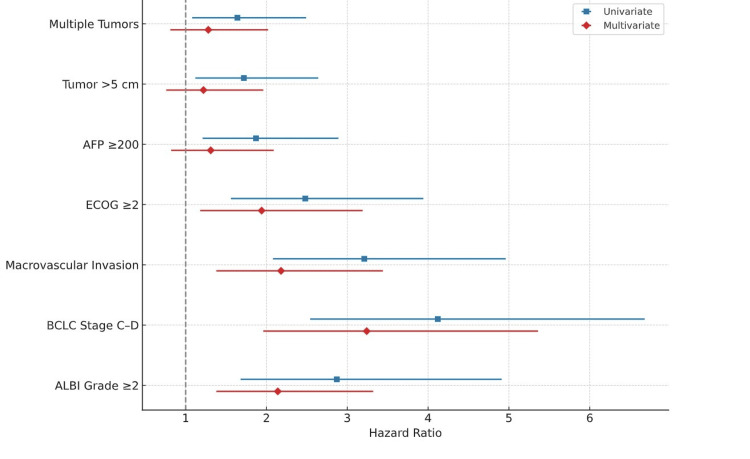
Prognostic factors for overall survival. Forest plot showing univariate and multivariate Cox regression analyses; ALBI grade, vascular invasion, BCLC stage, and ECOG performance status were independent prognostic factors. AFP, alpha-fetoprotein; ALBI, Albumin-Bilirubin; BCLC, Barcelona Clinic Liver Cancer; ECOG, Eastern Cooperative Oncology Group.

In multivariable analysis, ALBI grade remained an independent prognostic determinant (HR: 2.14, p < 0.001), while CP class did not retain significance (p = 0.171). BCLC stage, macrovascular invasion, and ECOG performance status also remained independently predictive of OS (Table 5, Figure 6).

**Table 5:** Univariate and multivariate Cox regression analysis for overall survival. AFP, alpha-fetoprotein; ALBI, Albumin-Bilirubin; BCLC, Barcelona Clinic Liver Cancer; CP, Child-Pugh; ECOG PS, Eastern Cooperative Oncology Group performance status; HR, hazard ratio.

Variable	Univariate HR (95% CI)	p-value	Multivariate HR (95% CI)	p-value
ECOG PS ≥2	2.48 (1.56-3.94)	<0.001	1.94 (1.18-3.19)	0.009
AFP ≥200 ng/mL	1.87 (1.21-2.89)	0.005	1.31 (0.82-2.09)	0.256
Multiple tumors	1.64 (1.08-2.49)	0.021	1.28 (0.81-2.02)	0.289
Tumor size >5 cm	1.72 (1.12-2.64)	0.013	1.22 (0.76-1.96)	0.408
Macrovascular invasion	3.21 (2.08-4.96)	<0.001	2.18 (1.38-3.44)	0.001
Extrahepatic metastasis	2.95 (1.78-4.89)	<0.001	1.67 (0.95-2.94)	0.076
BCLC stage C-D	4.12 (2.54-6.68)	<0.001	3.24 (1.96-5.36)	<0.001
CP-B vs. CP-A	2.38 (1.54-3.67)	<0.001	1.42 (0.86-2.35)	0.171
ALBI grade ≥2	2.87 (1.68-4.91)	<0.001	2.14 (1.38-3.32)	<0.001

The proportional hazards assumption was evaluated using Schoenfeld residuals, and no statistically significant violations were identified for either the global model or individual covariates (all p > 0.05), supporting the validity of the Cox proportional hazards models.

Progression-free survival

Similar trends were also apparent for progression-free survival. The median PFS was 11.8 months (95% CI: 9.4-14.6) for ALBI-1, 8.2 months (95% CI: 6.5-10.1) for ALBI-2, and 4.1 months (95% CI: 2.3-6.2) for ALBI-3 (log-rank p<0.001). The median PFS was 9.8 months for CP-A compared to 6.4 months for CP-B (log-rank p=0.004). The ALBI score demonstrated better discrimination for PFS with a C-index of 0.64 compared to 0.58 for the CP score (p=0.041).

## Discussion

The present retrospective analysis from a tertiary cancer center in North India demonstrates that the ALBI score provides significantly superior prognostic discrimination compared with the widely used CP classification in patients with HCC. This study, comprising 210 patients with extended follow-up to June 2025, not only confirms the prognostic value of ALBI reported in international cohorts but also addresses the paucity of data from the Indian subcontinent, where etiology, disease biology, and treatment patterns differ from Western and East Asian populations.

HCC poses a unique clinical challenge due to its dual pathology, where malignancy coexists with chronic liver disease or cirrhosis in the majority of patients [4,5]. Accurate assessment of hepatic reserve is therefore essential for treatment selection, prognostication, and estimation of risk of treatment-related toxicity. For decades, the CP score has remained the cornerstone of liver function assessment in HCC [6,7]. However, the limitations of CP-particularly the inclusion of subjective parameters such as ascites and encephalopathy-have been well recognized. These clinical features vary between observers and may fluctuate with medical therapy, leading to inconsistent classification and prognostic inaccuracy [9]. Furthermore, CP-A patients, who comprise the majority of candidates for curative or locoregional therapies, represent a heterogeneous population with variable outcomes that CP is unable to differentiate [10].

The ALBI score, proposed by Johnson et al. in 2015, was designed as an objective, laboratory-based method to evaluate hepatic function using only serum albumin and bilirubin [11]. Multiple international validation studies across heterogeneous treatment groups-including resection, ablation, TACE, and systemic therapy-have demonstrated that ALBI offers superior or comparable prognostic performance to CP and provides distinct stratification among CP-A patients [13-17]. The modified ALBI (mALBI) grade, which further subdivides ALBI-2 into grades 2a and 2b, has been shown to enhance prognostic differentiation [12].

Our results align closely with these global findings. ALBI and mALBI scores demonstrated clearer separation of overall survival curves than CP classification, with statistically significant differences across all ALBI grades. ALBI-1 patients achieved the best outcomes, while ALBI-3 patients had markedly reduced survival, reflecting progressively worsening hepatic reserve. In contrast, CP classification-especially within CP-A-showed limited discriminatory capability. Importantly, ALBI significantly differentiated prognosis among CP-A patients in our study, revealing a clinically meaningful 6.3-month difference in median overall survival between ALBI-1 and ALBI-2 subgroups. This supports the argument that CP-A encompasses diverse hepatic function levels that CP scoring does not adequately capture.

International studies echo these observations. Several primary studies evaluating systemic therapy have demonstrated superior prognostic performance of the ALBI score compared with the Child-Pugh score [4-5,9]. Diaz Romero et al. also reported significant differences in survival across ALBI grades in a Mexican HCC cohort, independent of CP score [4]. Lee et al. found ALBI to be predictive of early recurrence after curative resection, outperforming CP [5]. Similarly, Tada et al. demonstrated that mALBI was a superior prognostic classifier in unresectable HCC treated with lenvatinib [9]. Our findings in an Indian population, therefore, extend and validate these results in a different epidemiological and etiological context.

Several factors explain ALBI’s superior prognostic performance. ALBI uses continuous variables that reflect fundamental hepatic functions-albumin synthesis and bilirubin excretion-providing high sensitivity to mild changes in liver reserve. In contrast, CP converts continuous data into categorical scores, reducing granularity and compressing biological variability. Moreover, ALBI excludes subjective components, ensuring reproducibility even in multicenter settings, where consistent assessment of ascites and encephalopathy can be challenging [27].

ALBI was also independently associated with overall survival in multivariable analysis, while CP lost statistical significance once ALBI and other covariates were included. This suggests that ALBI captures the prognostic information inherent to CP more precisely. Modified ALBI demonstrated even stronger discriminatory performance-the highest C-index among all scoring systems tested-underscoring its utility, particularly in patients classified as ALBI-2, who represent the largest proportion of HCC cases worldwide and in our study [12].

Our study included patients across the full spectrum of BCLC stages and treatment modalities, reflecting real-world clinical practice. The prognostic value of ALBI was retained across these subgroups, supporting its broad applicability in HCC management. Tumor-related factors such as macrovascular invasion, extrahepatic spread, and advanced BCLC stage remained strong predictors of survival, consistent with established literature [24]. Nevertheless, ALBI provided additional discriminatory value independent of these tumor-related prognostic markers, reinforcing the importance of accurate liver function assessment as a parallel determinant of prognosis.

These findings have multiple clinical implications. First, ALBI can refine treatment decisions, particularly in selecting candidates for resection, TACE, stereotactic body radiotherapy (SBRT), or systemic therapy, where liver tolerance is crucial. ALBI-1 patients, for instance, may be considered for more aggressive or combination therapies, while ALBI-2b or ALBI-3 patients may require greater caution to avoid hepatic decompensation. Second, ALBI may improve clinical trial design and eligibility criteria. Many pivotal HCC trials have relied on CP-A status, inadvertently including patients with significantly different hepatic reserves [28]. ALBI offers a more consistent and objective stratification tool that could standardize trial populations and reduce confounding related to liver function [28,29]. Third, ALBI can potentially be used for dynamic monitoring. Early changes in ALBI during treatment have been shown to correlate with outcomes in systemic therapy settings [1], suggesting a role for serial ALBI assessments to evaluate hepatic tolerance and anticipate toxicity.

Our findings also lend support to evolving staging systems that incorporate ALBI. The modified BCLC framework and the Hong Kong Liver Cancer (HKLC) system have proposed ALBI as a more refined hepatic assessment tool than CP [30,31]. Adoption of ALBI may therefore enhance prognostic accuracy in both clinical practice and population-based research.

Despite its strengths, this study has several limitations. The retrospective, single-center design may have introduced selection and information bias, thereby limiting the generalizability of the findings. Although our sample size is relatively large for an Indian HCC cohort, several subgroup analyses, particularly those involving Child-Pugh subclasses and ALBI grade 3 patients, were based on relatively small sample sizes and should therefore be considered exploratory and interpreted cautiously until validated in larger prospective multicenter cohorts. Heterogeneity in treatment modalities, while reflective of real-world clinical practice, may have influenced survival outcomes despite multivariable adjustment, and residual confounding from unmeasured variables cannot be excluded. Additionally, because several subgroup and pairwise comparisons were exploratory, no formal correction for multiple testing was applied, and these findings should be interpreted with appropriate caution. Furthermore, the reported Harrell's C-index values represent apparent (in-sample) estimates and were not internally validated using bootstrap resampling; therefore, some degree of optimism in model performance cannot be excluded, underscoring the need for external validation in independent cohorts. Although follow-up was sufficient for meaningful survival analysis, longer-term outcomes, particularly among patients with early-stage disease, may not yet be fully captured. Finally, while the ALBI score is an objective measure of hepatic reserve, serum albumin and bilirubin concentrations may be influenced by intercurrent infection, nutritional status, or cholestasis unrelated to underlying liver function.

Nonetheless, the consistency of our findings with international data reinforces the robustness and generalizability of the ALBI score. Prospective, multicenter Indian cohorts with standardized treatment protocols and dynamic ALBI monitoring would further strengthen the evidence. Combining ALBI with emerging biomarkers or imaging-based assessments such as liver stiffness may yield even more accurate prognostic tools [18,19].

In summary, this study supports the incorporation of ALBI into routine clinical assessment for HCC in India. ALBI provides objective, reproducible, and superior prognostic stratification compared with CP, particularly within CP-A patients. Its integration into clinical decision-making pathways and clinical trial frameworks has the potential to improve treatment selection, risk stratification, and overall patient care.

## Conclusions

The ALBI score demonstrated superior prognostic discrimination compared with the Child-Pugh score in this retrospective single-center cohort of patients with hepatocellular carcinoma, particularly among those with preserved liver function (Child-Pugh class A). As an objective and reproducible measure of hepatic reserve, the ALBI score may provide incremental value for risk stratification and prognostic assessment in routine clinical practice. However, these findings should be interpreted within the context of the retrospective single-center study design, and larger prospective multicenter studies with external validation are warranted before widespread incorporation of the ALBI score into routine clinical decision-making.
